# Deep Neural Network Framework Based on Word Embedding for Protein Glutarylation Sites Prediction

**DOI:** 10.3390/life12081213

**Published:** 2022-08-10

**Authors:** Chuan-Ming Liu, Van-Dai Ta, Nguyen Quoc Khanh Le, Direselign Addis Tadesse, Chongyang Shi

**Affiliations:** 1 Department of Computer Science and Information Engineering, National Taipei University of Technology (Taipei Tech), Taipei City 106, Taiwan; 2 Samsung Display Vietnam (SDV), Yen Phong Industrial Park, Bac Ninh 16000, Vietnam; 3 Professional Master Program in Artificial Intelligence in Medicine, College of Medicine, Taipei Medical University, Taipei City 106, Taiwan; 4 Institute of Technology, Debre Markos University, Debre Markos P.O. Box 269, Ethiopia; 5 School of Computer Science and Technology, Beijing Institute of Technology, Beijing 102488, China

**Keywords:** glutarylation site prediction, deep neural networks, word embedding, LSTM, ELMo, GloVe

## Abstract

In recent years, much research has found that dysregulation of glutarylation is associated with many human diseases, such as diabetes, cancer, and glutaric aciduria type I. Therefore, glutarylation identification and characterization are essential tasks for determining modification-specific proteomics. This study aims to propose a novel deep neural network framework based on word embedding techniques for glutarylation sites prediction. Multiple deep neural network models are implemented to evaluate the performance of glutarylation sites prediction. Furthermore, an extensive experimental comparison of word embedding techniques is conducted to utilize the most efficient method for improving protein sequence data representation. The results suggest that the proposed deep neural networks not only improve protein sequence representation but also work effectively in glutarylation sites prediction by obtaining a higher accuracy and confidence rate compared to the previous work. Moreover, embedding techniques were proven to be more productive than the pre-trained word embedding techniques for glutarylation sequence representation. Our proposed method has significantly outperformed all traditional performance metrics compared to the advanced integrated vector support, with accuracy, specificity, sensitivity, and correlation coefficient of 0.79, 0.89, 0.59, and 0.51, respectively. It shows the potential to detect new glutarylation sites and uncover the relationships between glutarylation and well-known lysine modification.

## 1. Introduction

Post-translational modifications (PTMs), such as methylation, acetylation, glycosylation, ubiquitination, and phosphorylation, are chemical modifications that play a critical role in the functional diversity and complexity levels of promotes following the protein biosynthesis by regulating localization activity and interactions with other cellular molecules in most of the biological processes [1]. These modifications may occur at any time during the life cycle of a newly synthesized protein. Therefore, the identification and characterization of PTMs become a challenging task for a comprehensive understanding of cellular proteins and human diseases and provide extensive applications. As a prevalent and significant post-translational modification, lysine glutarylation has recently drawn a great deal of attention due to its involvement in diverse physiological and biological processes, including amino acid metabolism, fatty acid metabolism, and cellular respiration [2]. It is a type of lysine acyl modifications that contain malonylation, succinylation, and glutarylation. Lysine glutarylation itself is a protein PTM that can be regulated by SIRT5, a major enzyme in the cells. The SIRT5 catalyzes lysine deglutarylation both in vitro and in vivo and also reserves the glutarylation of carbamoyl phosphate synthetase 1 (CPS1) inhibits its activity [3]. In addition, enzymes such as thiolase, 3-hydroxy-3-methylglutaryl-coenzyme A (HMG-CoA) synthase, HMG-CoA lyase, d(−)-*β*-hydroxybutyrate dehydrogenase (bOHB), 3-hydroxy-3-methylglutaryl-CoA synthase 2 (HMGCS2), CPS1, and manganese superoxide dismutase (MnSOD) can participate in a variety of important enzymatic reactions [4]. It also plays an essential role in human sperm maintaining sperm motility [5]. The previous works suggested that glutarylation sites have been correlated to a lot of human diseases such as diabetes [6], glutaric academic type I disease [7], neuronal anaplerosis [8], and heart disease [9].

Prediction of PTM sites as well as lysine sites have been common in bioinformatics fields and there have been many studies conducted with promising performance [10]. Glutarylation found on lysine residues has revealed as an important regulator of several metabolic and mitochondrial processes [11]. However, little attention has been paid to enhancing glutarylation sites prediction and become a challenging task accordingly. GlutPred [12] is the first computational prediction of glutarylation sites in which they encoded mRNA codon-triplet as features. Next, iGlu-Lys [13] adopted a conventional machine learning support vector machine on amino acid pair order and special-position information to improve the predictive performance from GlutPred. In an effort to incorporate different features, AL-barakati et al. [14] implemented RF-GlutarySite based on a random forest classifier to predict glutarylation sites with independent test accuracy reaching 72%. Finally, a recent predictor for this purpose has been released by Huang et al. [15] in which they included intrinsic interdependence between positions in the substrate sites to improve the performance. At the end, their predictor could reach an accuracy of 71% on a benchmark independent dataset. Despite some positive achievements that have been made for the identification purpose in recent years, improvements are still needed to enhance glutarylation prediction. For example, multiple tools purposed for this prediction obtained undesirable performances, the low correlation coefficient between true and predicted values in comparison with prediction tools for other PTM sites.

According to recent studies, bio-sequence has been proven to be used for a broad range of bioinformatics research, such as family classification, visualization of proteins, prediction of structure, disordered recognition of proteins, and protein interactions [16], [17]. One of the main challenges for protein sequence analysis is contextualizing the structural properties of the desired proteins from the amino acid sequence database. An automated processing framework working effectively and optimizing time-consuming is essential for sequence data analysis. The advances in deep learning approaches applied in protein analysis have shown promising results and advantages in processing sequential data [18]. There has been growing evidence that deep learning approach can be successfully applied in protein prediction and genomic analysis [19]. There has existed a contextual relationship among amino acid sequences that biological sequences, particularly protein sequences, are comparable with natural language in terms of composition. Therefore, natural language processing (NLP) techniques have been used to address biological sequence processing [20]. Moreover, word embedding techniques widely used in NLP can be adopted to transform the contextual relationship among amino acid sequences. The integration of embedding techniques into deep learning enables us to solve biological sequence feature representation and extraction. In bioinformatics, word embedding techniques have been used to analyze the protein structural properties from its amino acid sequence representation learning [21,22]. In a similar way, the CNN-BiLSTM model has been used to identify and achieve more functionality than traditional models of the potential contextual relationships of amino acid sequences [23]. A new way of representing protein sequences as continuous vectors were proposed as a new biological language model, which effectively traces the biophysical properties of protein sequences from unlabeled big data (UniRef50) [24]. Based on the aforementioned studies, it would be reasonable to suggest that a deep neural network approach based on embedding techniques has great potential applications for glutarylation sites prediction.

In this study, we first conducted a thorough survey considering the state-of-the-art computational prediction tool, in which the algorithms, feature selection techniques, performance evaluation methods, and so on were meticulously discussed. In addition, we designed a novel Deep Neural Network framework based on word Embedding techniques (DNN-E) for protein glutarylation site prediction. The results show that our proposed framework could generate better optimal features for this problem, thus improving the performance by reducing the feature dimension as well as accuracy and confidence. This paper summarizes major contributions as follows:

(1) Develop a novel deep neural network framework for glutarylation prediction based on word embedding techniques;
(2) Evaluate the effectiveness of conventional machine learning, deep neural network models, including long short-term memory (LSTM), stacked LSTM (S-LSTM), bidirectional LSTM (B-LSTM), convolutional neural network LSTM (CNN-LSTM), and convolutional neural network bidirectional LSTM (CNN-BLSTM) in glutarylation prediction;
(3) Evaluate the prediction performance on different word embedding models, including embedding layer model, pre-trained word embedding techniques such as global vectors for word representation (GloVe), and embedding from language models (ELMo).

We organized the rest of this paper as follows: Section 2 shows the process material and methods, how to extract the glutarylation sites, and describes the overview architecture of the DNN-E framework, which shows a theoretical method of how to extract the glutalyration features and how to utilize the machine learning, deep learning techniques to classify the glutarylation sites. Section 3 provides the detailed experiment design and experimental results as well as the evaluation and comparison to the previous studies. Section 4 expands the discussion and the limitation. Finally, Section 5 draws conclusions and further study.

## 2. Materials and Methods

### 2.1. Data Preparation 

**Figure 1:**
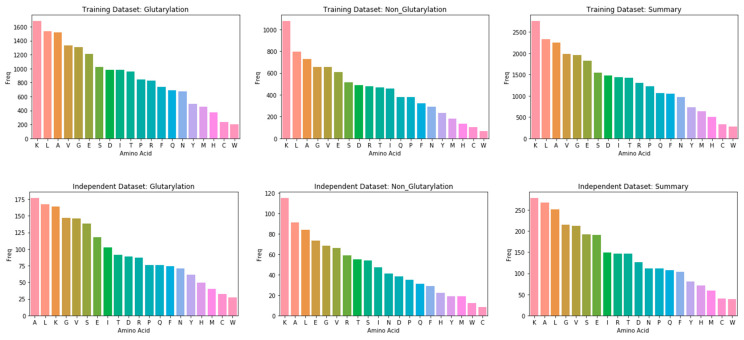
Amino acids distribution in the training set and independent test set. (The number of colors represents the number of amino acids).

In this study, glutarylation and non-glutarylation sites were extracted from MDDGlutar [15], which is the latest dataset for this prediction purpose. In this dataset, they firstly retrieved experimentally verified glutarylation sites from Protein Lysine Modifications Database (PLMD) [25]. After that, window sizes ranging from 11 to 25 were evaluated to select the best one. The cross-validation results showed that the 21-mer window size performed better than the other levels, thus they selected the 21-window size as an optimal one. For removing homologous sequence fragments, they then used CD-HIT software with a cut-off level of 40%. Finally, the rest of the data contains 522 glutarylation sites and 906 non-glutarylation sites as shown in Table 1. For validation purposes, the dataset was split into training and testing disjoint datasets with a ratio of 90% and 10%, respectively. The training set has been used to develop the model and the independent set will be used to evaluate the model performance. The detailed distribution of amino acids in the training and the independent test datasets are given in Figure 1.

### 2.2. Methodology

**Figure 2:**
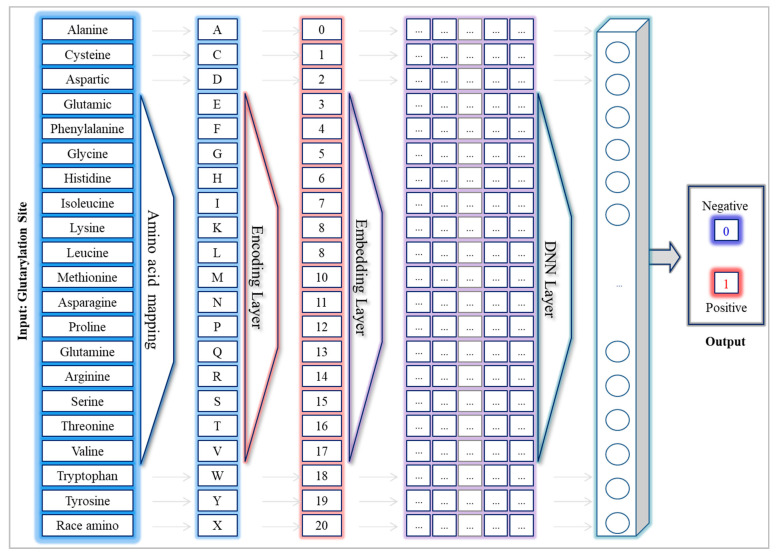
The overview architecture of the DNN-E framework.

Assuming S represents a sequential amino acid $x_{i}\;$ of a glutarylation site dataset: $S=\left\{x_{1},\;x_{2},\;\ldots ,\;x_{N}\right\}$, where N is the fixed length of each sequence. Each amino acid sequence has been classified into either positive or negative glutarylation. The objective of the proposed methodology is to conduct a DNN-E framework to perform prediction for the independent test set. In this section, we explain the detailed architecture of the DNN-E framework, and how to represent glutarylation site features. In general, the proposed DNN-E framework is composed of four layers: the encoding layer was used to encode each amino acid as an identical number; the word embedding layer was responsible for translating the encoded amino acids into pre-fixed length continuous vectors; then deep neural network models were built to capture the contextual features of amino acid sequences to support sequence classification at the output layer. The overview architecture of the proposed framework is presented in Figure 2.

#### 2.2.1. Word Embedding Layer

Word embedding is a learned representation approach for words and documents, in which an individual word is represented by a predefined dense vector representation, instead of a sparse vector representation. The main benefit of the dense representations is the generalization capability that is able to capture the similarities among the similarity words [26]. Therefore, word embedding has been used extensively in language modeling and natural language processing applications, such as sentiment analysis, and text classification [27,28]. Word embedding models have shown to be more efficient than the bag-of-words models or one-hot-encoding schemes. In the traditional bag-of-words model, each word or amino acid was represented by a large sparse vector based on the occurrence of words within a document. In word embedding, by contrast, each word was represented by a dense continuous vector space. Word embedding methods study the relationship between sequential elements in a predefined fixed-sized real-valued vector represented for the vocabulary of the corpus. The most commonly used are the embedding layer, Word2Vec, Global Vectors for Word Representation (GloVe), Embedding from Language Models (ELMo), and Bidirectional Encoder Representation from Transformers (BERT).

##### Embedding Layer

An embedding layer for a certain NLP mission, such as document classification, is a word embedding technology used as an integral feature of a neural network model. The embedding layer $f:\left\{x_{i}|x_{i}\in S\right\}\to R^{n}$ acted as a parameterized function mapping a word or character to a fixed-size high-dimensional vector. The embedded vector was initialized with a random weight vector. The weight vectors were updated during the training of neural networks. For example, given a sequence of amino acids as a sequential input vector represented by S=[A,F, K,…,V, K, N] where the length of *S* is the number of the amino acids. Firstly, each amino acid in S was encoded into a unique integer. The encoded vector denoted as X=[0, 4, 8, …, 17, 8, 11]. The embedding layer f was performed on each element of X to generate the output embedded vector for each amino acid, as illustrated in Figure 2.

##### Word2Vec

Word2Vec is a mathematical tool to learn word integration from a text corpus efficiently. It was created initially as a solution to make embedding effective in neural network-based training and has since become the de facto norm for pre-trained word embedding. This standard has been implemented [29]. The work also consisted of the analysis of learned vectors and the investigation of vector mathematics on word representation. There are two algorithms inside Word2Vec for producing word vectors: Skip-gram predicts context words that are given target word based on the likelihood of each context word being maximized in the current center term, and CBOW predicts target word from a bag-of-words context. There are two moderately efficient training methods: hierarchical softmax and negative sampling [30]. Both Word2Vec and the embedding layer learn to represent words to meaningful feature representation. However, Word2Vec is an unsupervised-based technique that tries to group the vectors of similar words in collinear vector space based on the cosine similarity measure. The embedding layer, in contrast, is a supervised technique that aims to optimize the weights throughout the training model.

##### Global Vector for Word Representation (GloVe)

Before GloVe was developed for word representation, the most common methods for the learning of word vectors are count-based global matrix factorization (LSA) or latent semantic analysis (LSA) [31]. However, despite showing remarkable positive on word representation, these methods still have their problems: LSA underperformed on the word similarity task, indicating a sub-optimal vector space structure; while skip-gram performed positively on the analogy, but was unable to utilize the statistics of the corpus since training is performed on separate local context windows instead of on aggregated global word-word co-occurrence statistics from a corpus. Therefore, the GloVe model, an extension to the Word2Vec model, was designed for efficiently learning word distributed representation based on an unsupervised learning algorithm. GloVe integrates the global statistics of matrix factorization techniques, such as LSA, with the local context-based learning in Word2Vec. However, local context learning is defined by an explicit word context or word co-occurrence matrix using statistics across the whole text corpus instead of by window-based in the Word2Vec [32].

##### Bidirectional Embedding from Language Models (ELMo)

Recently, a new method of in-contextualized word incorporation has been suggested in the Language Model Embedding (ELMo) to explore the meaning and semantics of the word and how these uses differ from one language to another. Contrary to the previous approaches in the study of text-based text vectors, word vectors have studied the functions of a deep bidirectional language model, a pre-trained word embedded in a wide corpus of texts [33].

##### Bidirectional Encoder Representation from Transformers (BERT)

Unidirectional is a big problem for word display models, which restricts the flexibility of architectures to be used during pre-training. BERT was therefore designed to pre-train a bidirectional representation of the unlabeled text through a combined reverse and forward context in all layers. As a result, the pre-trained BERT model can only be optimized to construct state-of-the-art models for a wide range of tasks, including answering questions and language inferences, without significant task-specific modifications to the architecture [34].

While Word2vec and Glove techniques are context-independent, both ELMo and BERT techniques are context-dependent by taking into account the word order relationship. As a practical implication, Word2vec and GloVe vectors can be used to train with a large corpus directly for downstream tasks. However, in the case of ELMo and BERT, word embedding vectors are obtained by training a model unsupervised on a corpus. The differences are summarized in Table 2.

#### 2.2.2. Deep Neural Network Framework

**Figure 3:**
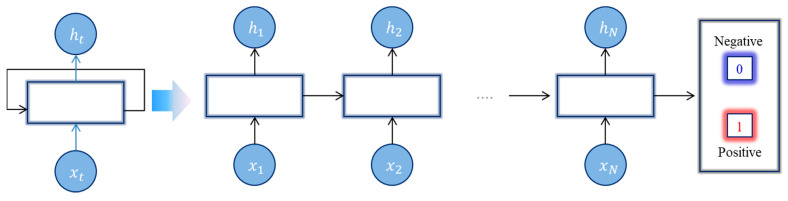
RNN architecture for sequence classification.

Deep learning algorithms have proven to be more efficient than traditional machine learning algorithms, which automate processes to solve complex tasks. A deep neural network (DNN) is an extension of a multi-hidden layer artificial neural network (ANN) which allows DNN to perform multiple sophisticated tasks, where each layer is connected only to the previous one and connected only to the next layer in the cached portion. The most common types of DNN architectures are recurrent neural networks (RNNs) and convolutional neural networks (CNNs). In some cases, RNNs and CNNs can be combined to utilize the benefit of DNN architecture known as hybrid deep neural networks. RNN is specialized for processing a sequence of values $x_{1},x_{2},\;\ldots ,\;x_{N}$ where the output of the current state $h_{t}$ depends on the output of the previous state $h_{t-1}$. RNN with recurrent connections between hidden units reads an entire sequence and then produces classification output, as illustrated in Figure 3. RNN models have been successfully implemented in the fields of NLP [35]. Unfortunately, RNNs suffer from the problem of vanishing gradients as processing long sequential data, in which the sensitivity of hidden states and outputs on a given input becomes weaker as moving along the sequence [36].

Long Short-Term Memory Network

**Figure 4:**
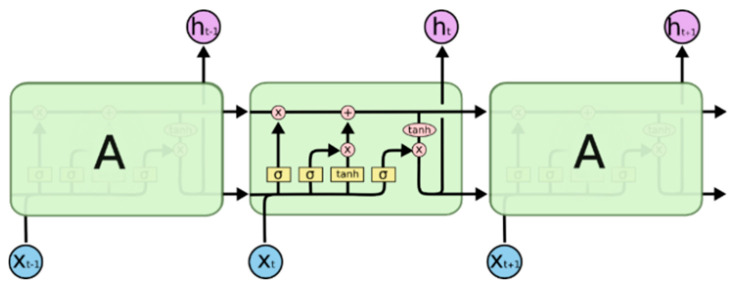
LSTM architecture [38].

A special variation of RNN built for sequence modeling is the Long Short-term Memory (LSTM) [37]. The multiplication gates improve the absence of the gradient problem by allowing LSTM cached states to store and access over a longer period, which eliminates the negative impact of the vanishing gradients issue. LSTM architecture is shown in Figure 4. There are three gates: *forget, input, and output gates*, in which the *forget gate* controls how much information the memory cell conveyed from the memory cell from the previous step; the *input gate* specifies whether the memory cell will be updated. In addition, it also controls how much information the current memory cell will receive from a potentially new memory cell; the *output gate* controls the value of the next hidden state. Suppose $x_{t}$ is the input vector at the time step t, $h_{t-1}$ denotes the hidden state at time step *t* − 1, $i_{t}$ denotes the input gate at the time step t, ${\hat{C}}_{t}$ denotes the candidate values to be added to input gate output at the time step t, $b_{i}$ and $b_{C}$ denotes the bias of the input gate and the candidate value, $W_{i}$ and $W_{C}$ denote the weights of the input gate and the candidate value computation. The input gate decides which values will be updated into the cell state.

(1) $$i_{t}=\sigma \left(W_{i}\left[h_{t-1},x_{t}\right]+b_{i}\right)$$

(2) $${\hat{C}}_{t}=\tanh\left(W_{C}\left[h_{t-1},x_{t}\right]+b_{C}\right)$$

The output vectors traversed the network between consecutive time steps t, t+1 is denoted by $h_{t}$. The multiplicative gates are the key to updating and controlling the cell states. It looks at $h_{t-1}$ and $x_{t}$, and outputs a value between 0 and 1 via a sigmoid unit for each number in the cell state $C_{t-1}$. Then, a tanh layer calculates vector of new candidate values, $C_{t}$ that could be added to the state. The output gate $\;o_{t}$ is responsible for the output based on the input and the memory in the cell being sent to the network as the input of the following time step $h_{t}$.

(3) $$C_{t}=f_{t}\times C_{t-1}+i_{t}\times {\hat{C}}_{t}\;$$

(4) $$f_{t}=\sigma \left(W_{f}\left[h_{t-1},x_{t}\right]+b_{f}\right)\;$$

(5) $$o_{t}=\sigma \left(W_{0}\left[h_{t-1},x_{t}\right]+b_{0}\right)\;$$

(6) $$h_{t}=o_{t}\times tanh\left(C_{t}\right)\;$$

##### Bidirectional Long Short-Term Memory Network (B-LSTM)

**Figure 5:**
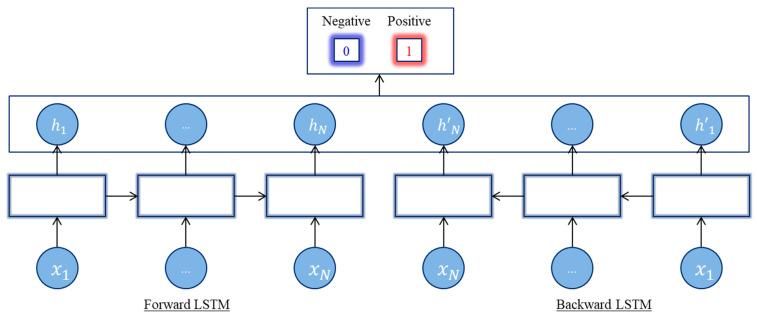
Bidirectional LSTM architecture.

The B-LSTM is the traditional LSTM variance that can improve sequence classification performance. The B-LSTM aggregates two reversed unidirectional LSTMs, thus providing the network with a further context and providing faster learning and even more complete information on the problem, as illustrated in Figure 5. B-LSTM was used for the management of long sequences of pseudo proteins and a better collection of subsequent reliance information [39].

##### Stacked Long Short-Term Memory Network (S-LSTM)

**Figure 6:**
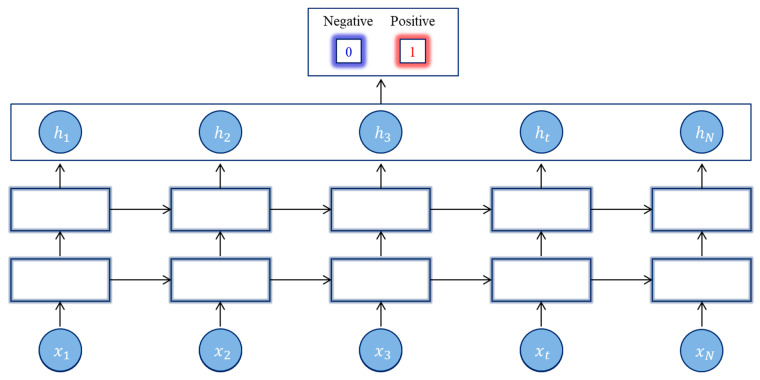
Stacked LSTM architecture.

S-LSTM is also another LSTM extension model, using multiple hiddenLSTM layers where each layer contains multiple memory cells, as shown in Figure 6. In this sense, the DNN can be interpreted as a pipeline processing architecture where the output is provided by each layer for a part of the assignment, and parameters are shared in the next layer [40]. The additional overshadowing layers will improve the learned image using previous layer results and create a new image at a high abstract level. The depth of DNN increases as well as the need for a sufficiently deep additional hidden layer and fewer neurons.

##### Hybrid Deep Neural Networks

**Figure 7:**
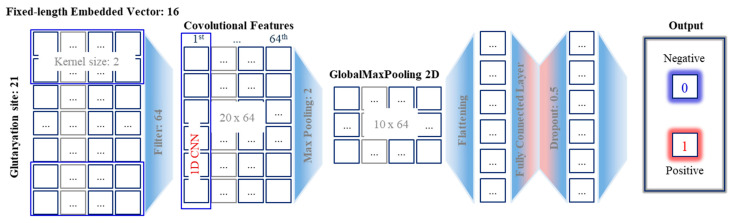
1D CNN architecture.

A convolutional neural network (CNN) is a specialized kind of neural network and was initially designed for image processing and analysis [41]. However, it has been widely used in natural language processing [42,43]. Instead of taking an image pixel matrix, sentences are taken as inputs of NLP tasks [44]. Each word of the sentence corresponds to a fixed-length embedded vector. For instance, suppose that each amino acid is represented by a 16-dimensional vector for a sequence amino acid comprised of 21 amino acids, therefore, the size of the input matrix will be [16 × 21]. One-dimensional CNN (1D CNN) works effectively with learning features from shorter segments of the overall dataset, in which the location of the feature within the segment is not high relevance. Kernel size is the size of the sliding window that convolves across the data. The filter or feature detector defines how many sliding windows going to run through the data. Suppose there are 64 filters and 2 rows as the kernel size. Max pooling was used to prevent overfitting of the learned features by taking the max value of multiple features based on the configure sliding window. As illustrated in Figure 7, convolutional refers to a filter, each filter sees 2 amino acids at a time step. One-dimensional CNN will perform the filter on entire input for each word vector. Padding was also considered to use to guarantee all the output has a fixed length. The output for one filter is a vector size of 20. Therefore, the 1D CNN output is the size of the [20 × 64] matrix. In the max-pooling layer, we perform the max operation on the output of each filter over the sentence.

The extracted spatial features from 1D CNN can be learned as sequences by an RNN such as LSTM, and BLSTM. Max-pooling layers are used to extract the most important features from the embedding layer and then feed the consolidated features to the LSTM. The pooling layer can use the standard length to halve the feature map size. The combination of 1D CNN and RNN models requires a particular design since each model has a specific architecture and its properties. While CNN is known as suitable for spatial feature extraction, LSTM and BLSTM keep the chronological order between amino acids in a sequence, thus it can ignore unnecessary elements using the forget gate. 

The last dense layer refers to a fully connected network layer, which provides learning features from all the combinations of the previous layers. Sigmoid activation function was used to produce a probability distribution over the 2 output classes. The final output layer consists of 2 neurons including its probability. All probabilities add up to 1.

## 3. Experiment and Results

This section describes the experimental settings and the selected parameters of the proposed DNN-E framework for glurarylation prediction. In addition, it presents a performance evaluation metric that was used in the validation and performance comparison.

### 3.1. Experimental Setup 

All experiments were conducted using Python 3.7, Keras library with TensorFlow backend [45], and Adam optimization. An Intel Core i7-7700 (3.60 GHz) CPU with 64 GB of memory was used with the CenOS Linux machine supported GeForce GTX 1080 Ti 11176 of memory GPU. In the beginning, a k-fold cross-validation approach [46] was used in this study. The training set was first randomly partitioned into five equally size portions or folds. Subsequently, four out of five portions of the training set were used to train while the remaining one-fifth of the training set was used to validate the performance of the training model.

#### 3.1.1. Embedding Layer Parameters

The embedding layer was constructed as the first hidden layer of the DNNs in which the embedding was learned along with multiple deep learning models. There are three prerequisite arguments used to construct the embedding layer, which is the first hidden layer of the DNN. First, the size of vocabulary known as the input dimension interpreted the total unique amino acids in the dataset. There are 20 amino acids and 1 rare amino acid, which are combined in different ways to make protein. Therefore, the size of vocabulary should be selected as 21. However, in order to avoid a collision, the size of vocabulary 30 is chosen. Secondly, the size of the vector space equal indicates the size of the output feature dimension in which amino acids will be embedded. The size of output vectors could be 8, 16, or higher. In this case, the size of output dimension 16 was found to be the most efficient result. Finally, the input length refers to the size of the input glutarylation site. In this work, all the selected glutarylation sites have a fixed size of 21, therefore, the input length of 21 is chosen. As shown in Table 3, there were three required input parameters of the DNNs.

#### 3.1.2. GloVe, ELMo Embedding Parameters

The pre-trained word embedding dataset is given by GloVe [47]. It was trained on one billion tokens with a 400-thousand-word vocabulary. Some embedding vector sizes are available, of which 50, 100, 200, and 300 are included. We used the GloVe 100 dimensions to train the model by integrating the vector scale. The embedded output vectors have been used in the embedded layer portion as the data for CNN-LSTM ‘s deep training model.

ELMo vector assigned to a token or amino acid is a function of the entire sequence, unlike traditional word embedding techniques such as Word2vec and GLoVe. Therefore, the same symbol would have different embedded vectors under different contexts. ELMo embedding module available in TensorFlow-hub. Each sequence of amino acids was tokenized into a list of characters before fitting into the ELMo embedding model. Table 4 summarizes the detailed selected parameters used for pre-trained embedding models. 

#### 3.1.3. Deep Neural Networks Hyperparameters

Most deep learning algorithms offer various hyperparameters that control multiple aspects, such as time consumption, computational resources, and accuracy of the algorithm. Hyperparameter tuning refers to the process of fine-tuning the optimal setting values in order to achieve the lowest generalization error and adjust the effective capacity of the model subject to computational resources. In order to configure the optimal hyperparameters for each training model, we have thoroughly evaluated the performance of those models on different measurements. For example, the number of neurons, the number of batch size, and the number of epochs were between 50 to 200 neurons, 25 to 100 epochs, and 10 to 30 batch sizes, respectively. The dropout rate was also used in each model to eliminate the overfitting effect. The final optimal hyperparameters are shown in Table 5.

1. Number of neurons characterizes the dimensions of hidden stages (outputs);
2. Number of epochs specifies the number of times that the learning algorithm will work through the entire training dataset;
3. Number of batch sizes defines the number of samples to work through before updating the internal model parameters. The size of a batch must be more than or equal to one and less than or equal to the number of samples in the training dataset;
4. Dropout is a regularization technique that decides the probability of a network neuron or node being excluded from activation and weight updates while training a network. The dropout rate shows the effect of reducing overfitting and improving model performance;
5. Kernel size or convolution filter is a hyperparameter used in a convolutional neural network. Kernel size determines the size of the sliding window that convolves across the data. The filter or feature detector defines how many sliding windows going to run through the data.

### 3.2. Performance Evaluation

Different statistical scores as defined in [48,49] have been used to assess each classification’s performance. Each query point in the test sets has its true class label in a usually supervised binary classification problem. 

The classifier maps the question points in one of the categories during the evaluation process: True Positive (TP), True Negative (TN), False Positive (FP), and False Negative (FN). In this method, the problem is a positive or negative concern for a specific class. On this basis, each class is determined by TP, TN, FP, and FN. To assess the output of the classifier, the following statistical results are used for each class:

Accuracy (ACC)

(7) $$\mathrm{ACC}=\frac{\mathrm{TP}+\mathrm{TN}}{\mathrm{TP}+\mathrm{FP}+\mathrm{TN}+\mathrm{FN}}$$

Recall/Sensitivity (SN)

(8) $$\mathrm{SN}=\frac{\mathrm{TP}}{\mathrm{TP}+\mathrm{FN}}$$

Specificity (SP)

(9) $$\mathrm{SP}=\frac{\mathrm{TN}}{\mathrm{TN}+\mathrm{FP}}$$

Matthew’s correlation coefficient (MCC)

(10) $$\mathrm{MCC}=\frac{\mathrm{TP}\;\times \;N-\mathrm{FP}\;\times \;\mathrm{FN}}{\sqrt{\left(\mathrm{TP}+\mathrm{FP}\right)\left(\mathrm{TP}+\mathrm{FN}\right)\left(\mathrm{TN}+\mathrm{FP}\right)\left(\mathrm{TN}+\mathrm{FN}\right)}}$$

Both sensitivity and specificity are appropriate for evaluating classification models for most datasets because these measures consider all entries in the confusion matrix. While sensitivity deals with True Positives and False Negatives, specificity deals with False positives and True Negatives. In other words, the combination of sensitivity and specificity is a comprehensive measure when both true positives and true negatives should be considered. The Matthews correlation coefficient (MCC), instead, is a more reliable statistical rate that produces a high score only if the prediction obtained good results in all of the four confusion matrix categories (True Positives, False Negatives, True Negatives, and False Positives), proportionally both to the size of positive elements and the size of negative elements in the dataset. The Matthews correlation coefficient is in the range [−1,1] where values of −1 and 1 indicate the worst-possible and the best-possible classifier, respectively.

The receiver operating characteristics (ROC) curve indicates the probability of classifying between classes, especially commonly used as the main performance metric in binary classification evaluation. The curve provides a convenient diagnostic tool to investigate one classifier with different threshold values and the effect on the True Positive Rate and False Positive Rate. One might choose a threshold in order to bias the predictive behavior of a classification model. The receiver operating characteristics area under the curve (ROC-AUC) represents the degree or measure of separability. This single score can be used to compare binary classifier models directly. As such, this score might be the most commonly used for comparing classification models for imbalanced problems. The score is a value between 0 and 1 for a perfect classifier.

### 3.3. Experimental Results

#### 3.3.1. Model Selection 

**Figure 8:**
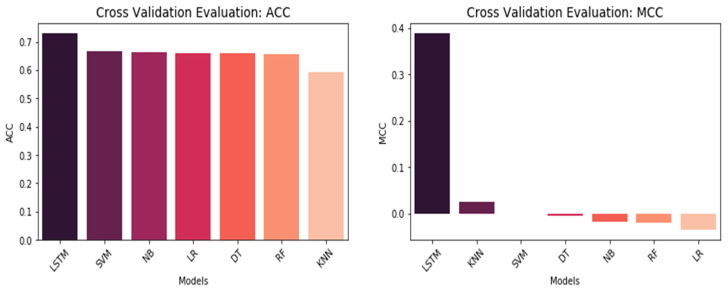
Cross-validation comparison on ACC and MCC for model selection.

Our first objective aims to evaluate the performance between the conventional machine learning models and the LSTM model based on the embedding layer for model selection. K-folds cross-validation process is illustrated in Figure 8 in which training dataset including 1290 glurarylation sites is split into *k* equally sized subsets called folds. One of the k-folds will act as the validation set, known as the holdout set, and the remaining folds are used to train the model. This process repeats until each of the folds has acted as a holdout fold. After each evaluation, a score is retained, and the average score represents the overall performance of the training model as all iterations have been completed. The independent test set includes 138 glurarylation sites separated from the training set and will only be used for testing to ensure that testing data have not been used in the training set. The results of cross-validation performance on the training test set are given in Figure 8. As can be seen, the LSTM model outperformed the conventional machine learning models by obtaining the highest ACC and MCC correlation coefficient. There is a noticeable gap in performance between LSTM and conventional machine learning models, which is a significant effect of the LSTM model. The accuracy rate obtained by the LSTM model was approximately 0.73 on average, while the accuracy rate was almost the same around 0.67 on average in conventional machine models. Furthermore, the MCC correlation coefficient rate obtained by the LSTM model signed a significantly higher 0.39 compared to conventional machine learning models producing even negative rates in some models such as Linear Regression (LR), Naive Bayes (NB), and Random Forest (RF) classifiers. The main reason for the unsatisfactory performance of the conventional machine learning model is that these models were only able to predict negative instances with higher specificity. In contrast, the negative prediction rate or specificity obtained by the LSTM model was significantly higher compared to machine learning models. It indicated that the predicted results obtained by the LSTM model based on the embedding layer were more accurate and reliable than the conventional machine learning models. Therefore, the results provide compelling evidence that the LSTM model enables to capture of the dependency relationship between amino acids in the sequence while making predictions. LSTM shares parameters (weights) update at each time step; therefore, the prediction task should be able to utilize the previously predicted results. The detailed results for 5-folds cross-validation are summarized in Table 6.

**Figure 9:**
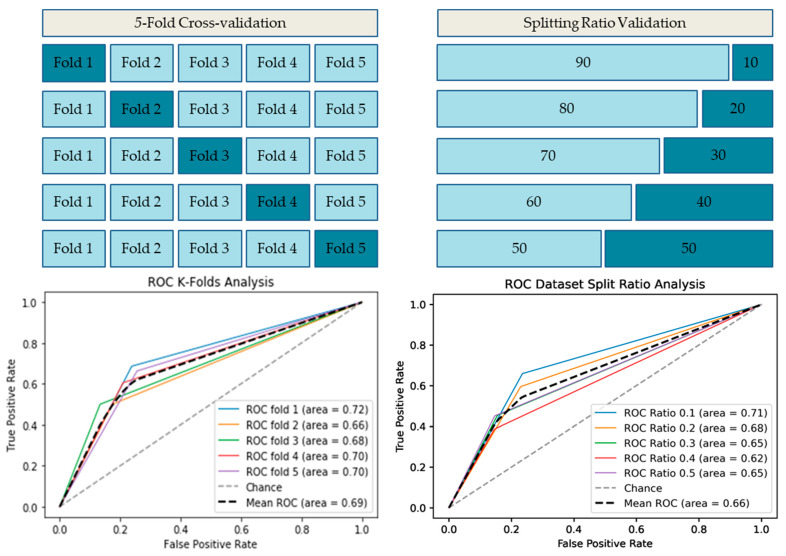
ROC-AUC plot for LSTM cross-validation.

**Figure 10:**
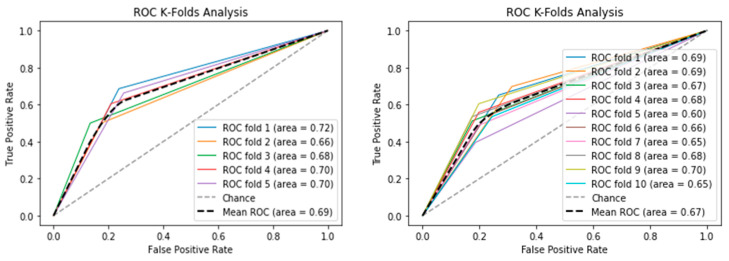
K-fold cross-validation comparison.

**Figure 11:**
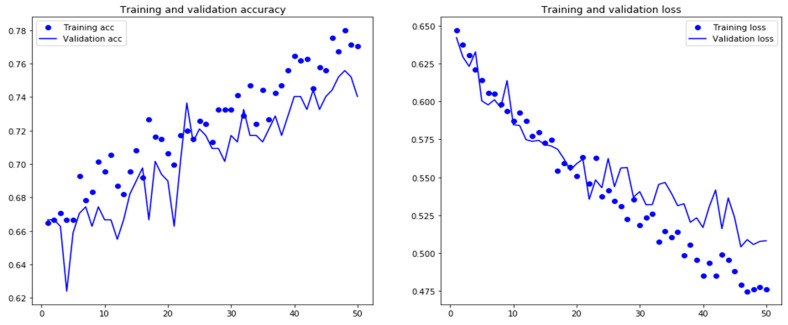
The accuracy and loss of training and validation sets for the LSTM model.

The receiver operating characteristics (ROC) plot was used to evaluate classification accuracy. While the area under the ROC (AUC) curve represents the capability of distinguishing between classes. Moreover, we also evaluated the training: testing the splitting ratio to select the most efficient splitting ratio. The 5-fold cross-validation procedure and splitting ratio evaluation results are shown in Figure 9. In 5-fold validation, the LSTM model obtained a high value of the AUC score with 0.69 on average. It indicates that our model achieves a highly reliable performance on unseen data. As shown in Figure 10, the training:testing splitting ratio 90:10 obtained the highest AUC score. As we compared the performance of the AUC score between 5-fold with 10-fold cross-validation, we identified that the AUC score of the 5-fold validation obtained a higher score on average than the 10-fold validation. The variation in training and validation accuracy and loss is shown in Figure 11. As the number of epochs increases, there is a probability of an overfitting problem occurring. 

#### 3.3.2. DNNs Variance Architecture Evaluation

**Figure 12:**
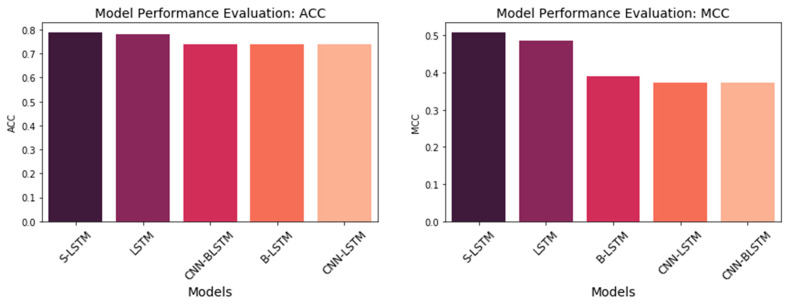
Performance comparison between DNNs.

**Figure 13:**
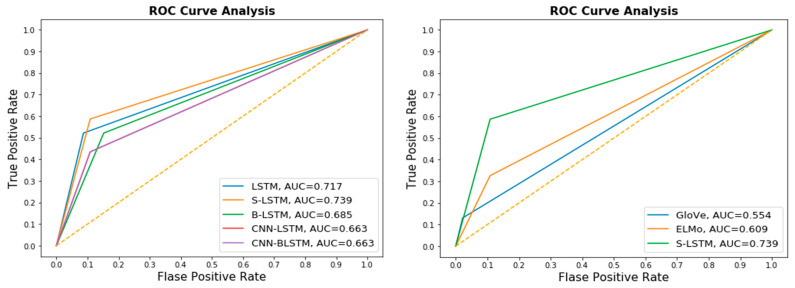
ROC curves of the DNNs (**left**) and pre-trained models (**right**).

**Figure 14:**
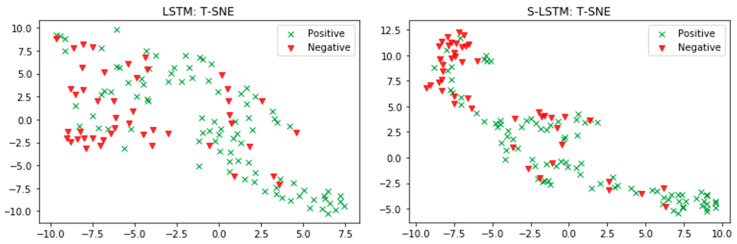
T-SNE visualization of LSTM and S-LSTM prediction.

Next, we evaluated the performance of the proposed DNN-E framework by replacing the LSTM model with the hybrid deep neural networks to reveal how the effectiveness of different models on amino acid sequence processing. The comparison performance between multiple DNN models based on the embedding layer for the independent test dataset is shown in Figure 12. As can be seen, the S-LSTM model obtained the highest performance both on accuracy score, and correlation coefficient scores (0.79:0.51). LSTM model obtained the same accuracy score but lower *MCC* correlation coefficient scores compared to the S-LSTM model. Although the correlation coefficient score obtained by the B-LSTM model was roughly equivalent to hybrid CNN-LSTM, and CNN-BLSTM models, the accuracy score was slightly higher. It can be observed that the LSTM model and S-LSTM performed better than the hybrid LSTM models. It can be interpreted that the feed-forward network is trained to learn the sequence of input more effectively compared to the spatial mapping on CNN. We also reasoned that adding recurrent network layers improves the performance of LSTM architecture. Hybrid CNN-LSTM and CNN-BLSTM models show less effectiveness in predictions compared to S-LSTM and LSTM models. Hybrid LSTM models such as CNN-LSTM, and CNN-BLSTM obtained the lower *ACC* and *MCC* (0.74:0.37). ROC-AUC analysis is given in Figure 13 as a comparison between these classifiers. S-LSTM proved that it obtained the highest AUC score. The detailed confusion matrix for independent testing is summarized in Table 7. The t-Distributed Stochastic Neighbor Embedding (TSNE) [50] was used to visualize the prediction results, as given in Figure 14.

#### 3.3.3. Word Embedding Techniques Evaluation 

**Figure 15:**
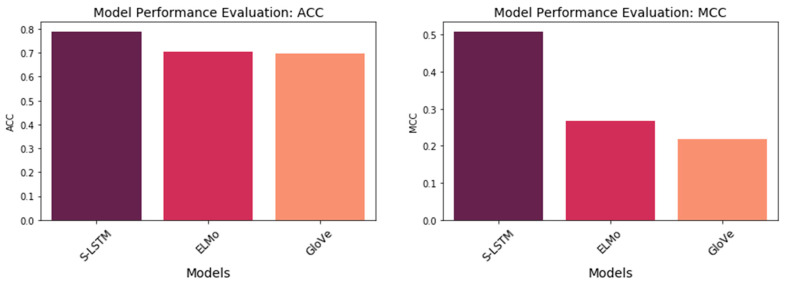
Performance comparison between S-LSTM to pre-trained word embedding models.

In order to evaluate the impact of word embedding techniques on prediction results, pre-trained word embedding datasets, including GloVe and ELMo were used to replace the embedding layer in DNN. Figure 15 shows the comparison between embedding layer performance and the pre-trained word embedding dataset. As can be seen, the S-LSTM model based on the embedding layer outperformed GloVe and ELMo models in both ACC and MCC. Although pre-trained word embedding was shown a significant impact on word representation, their performance was unattractive on the independent test set compared to the S-LSTM model based on the embedding layer. It can be interpreted that the pre-trained word embedding models could pick up more semantic signals in text processing. However, each amino acid has a unique function in the chain of the protein. The sequential positions of the amino acid are more important attributes needed to be captured. In other words, there is an existing hidden pattern of amino acid sequence of classification in which deep neural network and embedding layer work more effectively. Despite GloVe obtaining accuracy as the same as ELMo, it provided a lower MCC score in prediction in this case. The detailed results are given in Table 8. 

#### 3.3.4. Comparison with the Previous Research

For a comparison between our model and the previously published works on the same problem, we retrieved the performance results from the previous four works [12,13,14,15]. In these works, they only used machine learning techniques for lysine glutarylation sites by extracting different sets of features e.g., amino acid pair order and substrate sites. Although the LSTM model exhibited a lower SN score than integrated SVM (i-SVM) [15] in the 5-fold cross-validation phase, it obtained much higher scores for SP, ACC, and MCC, as listed in Table 9. The same results were observed using the independent test set. As shown in Table 10, our optimal model S-LSTM achieved a lot of improvements as compared to the other published works [12,15]. S-LSTM model obtained an SN score that was 9% lower compared to the i-SVM model obtained but 9% higher compared to the Gluted obtained. While S-SLTM obtained a much higher SP score compared to SP obtained by i-SVM. Interestingly, our proposed model outperformed with respect to ACC and MCC on the independent test set. It determines that we can find a novel set of signatures that might be more suitable for this classification purpose. Furthermore, the use of deep neural networks helps us generate a hidden feature set of features that makes our model more robust than the machine learning algorithms. 

## 4. Discussion

The pre-trained word embedding vector has a larger dimensional embedding vector and it shows a great effect on natural language processing such as sentiment analysis. The capture semantic of a word is one of the significant functions of the pre-trained embedding vector model. In other words, the same word in the token will have a different meaning or embedding value based on the position of the word. However, this strategy shows a limited effect on the amino acid sequence. Because each amino acid has a unique function in the chain. The order sequence of amino acids plays an important factor in identifying and classify the function of the protein. Therefore, capturing semantics in this scenario has little impact on classification results. Testing on large datasets could be performed in order to conclude this evaluation. In comparison with the longer natural language processing model with 1024 proteins ranging from about 30 to 33,000 residues [24]. Further GPU memory is required for longer proteins, and the underlying models can only keep a limited record of long-range dependence. Protein uses 20 standard amino acids in most cases and 5 additional characters in unusual, undefined, or unknown causes, compared to up to a limit of two million natural language processing terms. Less vocabulary could be problematic if protein sequences encode sentences of similar complexity. 

In this study, several deep learning models for protein function prediction (e.g., glutarylation sites) based on a variety of biological data forms are addressed, which analyzed the evolution of machine learning approaches used to predict protein function based on trained data. Although there was an increase in the usage of computational models to extract significant functions and create good-appearing predictors, techniques using deep learning strategies had been still capable of outperforming other methods. One of the challenges that deep learning faces is that it needs a large amount of data, which possibly limits its effectiveness, at least in certain research on predicting protein function. Several methods covered in this study achieved excellent findings over a diverse range of functional groups. However, several other methods that did not produce similar outcomes need to be discussed for a variety of reasons, including the following: (1) When additional data is available for training their models, their outcomes may improve; (2) Technology advancements may result in improved outcomes; and (3) By combining these techniques with a more effective one, we may be able to get better results than using them separately.

All supplementary glutarylation site datasets and optimal training models for this article can be found online downloaded at https://github.com/CSIE-NTUT/DNN-E (accessed on 26 June 2022). Our DNN-E framework built for online glutarylation sites prediction is available at https://share.streamlit.io/slime21023/bio_dnne/main/app.py (accessed on 26 June 2022).

## 5. Conclusions

In this study, we proposed a novel DNN-E framework for glutarylation prediction. We found that the deep neural network approach obtained higher accuracy and confidence rate than previous research. It is another compelling evidence to prove that, deep neural networks can work effectively with biological sequence data to handle complex problems in protein identification. The embedding layer added in deep neural network work more productively compared to pre-trained word embedding such as GloVe and ELMo. This study, therefore, indicates that word embedding, in general, provides a mechanism to transfer the language of biology. In addition, this work provides the potential to detect and detect new sites of glutarialisation and reveal the links between glutarial and well-known protein acetylation and methylation for modifications of lysine, including malogylation and succinylation recently identified. The small dataset for training is considered a limitation of model performance. The extension of work to optimize the framework should be conducted in further research. 

Nevertheless, scientists are now going to resort large amount of input features, especially those that have been taken from biological sequences. To close the gap between the known and unknown sequences, reliable data-driven models are essential. which would help to know the effects of protein mutations on illnesses and the development of novel proteins. Finally, we are convinced that an effective scientific procedure can be developed in which hypotheses are produced by applying the best method for predicting functions to the scientific data that is currently available. These theories are then put to the test in the lab, resulting in confident predictions of a protein’s function. We anticipate that the results of this study will be helpful to computational and laboratory molecular biology professionals and complete this mission more efficiently.

## Figures and Tables

**Table 1:** Dataset Description.

Instances	Training	Independent Testing	Dataset
Glutarylation	430	92	522
Non-glutarylation	860	46	906

**Table 2:** Word Embedding Techniques Comparison.

Word Embedding	Context Sensitive	Learned Representation
Word2Vec	No	Words
GloVe	No	Words
ELMo	Yes	Words
BERT	Yes	Sub-words

**Table 3:** Embedding layer parameters.

Embedding Layer	Parameters
Vocabulary size	30
The size of the vector space	16
The length of input sequences	21

**Table 4:** Pre-trained embedding model parameters.

Parameters	Pre-Trained Embedding	
	GloVe	ELMo
No. of neurons	100	100
No. of epochs	25	25
No. of batch size	10	10
Dropout rate	0.5	0.5
Input dimensions	100	1024

**Table 5:** DNNs Hyperparameters.

Hyperparameters	LSTM	S-LSTM	B-LSTM	CNN-LSTM	CNN-BLSTM
Neurons	100	100	100	100	100
Epochs	50	25	50	25	50
Batch size	10	10	10	10	10
Dropout rate	0.3	0.3	0.3	0.3	0.3
Filter: kernel size	-	-	-	64:5	64:5

**Table 6:** 5-folds cross-validation for LSTM model.

Dataset	TP	FP	TN	FN	SN	SP	ACC	MCC
Fold 1	59	41	131	27	0.69	0.76	0.74	0.43
Fold 2	43	30	142	43	0.50	0.83	0.72	0.34
Fold 3	43	23	149	43	0.50	0.87	0.74	0.40
Fold 4	52	36	136	34	0.60	0.79	0.73	0.39
Fold 5	57	44	128	29	0.66	0.74	0.72	0.39
**Average**	**50.8**	**34.8**	**137.2**	**35.2**	**0.59**	**0.80**	**0.73**	**0.39**

**Table 7:** Summary of DNNs performance on the independent test set.

Dataset	TP	FP	TN	FN	SN	SP	ACC	MCC
Fold 1	59	41	131	27	0.69	0.76	0.74	0.43
Fold 2	43	30	142	43	0.50	0.83	0.72	0.34
Fold 3	43	23	149	43	0.50	0.87	0.74	0.40
Fold 4	52	36	136	34	0.60	0.79	0.73	0.39
Fold 5	57	44	128	29	0.66	0.74	0.72	0.39
Average	50.8	34.8	137.2	35.2	0.59	0.80	0.73	0.39

**Table 8:** Pre-trained word embedding techniques performance comparison.

Models	TP	FP	TN	FN	SN	SP	ACC	MCC
**S-LSTM**	**27**	**10**	**82**	**19**	**0.59**	**0.89**	**0.79**	**0.51**
GloVe	6	2	90	40	0.13	0.98	0.70	0.22
ELMo	15	10	82	31	0.33	0.89	0.70	0.27

**Table 9:** Comparison between LSTM model to i-SVM model.

Cross-Validation	SN	SP	ACC	MCC
**LSTM**	**0.59**	**0.80**	**0.73**	**0.39**
i-SVM	0.68	0.62	0.64	0.28

**Table 10:** Performance comparison on the independent test set.

K_Average	TP	FP	TN	FN	SN	SP	ACC	MCC
**S-LSTM**	**27**	**10**	**82**	**19**	**0.59**	**0.89**	**0.79**	**0.51**
i-SVM	30	24	68	16	0.65	0.74	0.71	0.38
Gluted Tool	25	8	84	21	0.54	0.91	0.79	0.50
